# Experimental drought indirectly enhances the individual performance and the abundance of an invasive annual weed

**DOI:** 10.1007/s00442-020-04711-y

**Published:** 2020-07-17

**Authors:** Andrea Mojzes, Gábor Ónodi, Barbara Lhotsky, Tibor Kalapos, György Kröel-Dulay

**Affiliations:** 1 Institute of Ecology and Botany, MTA Centre for Ecological Research, Alkotmány u. 2-4, 2163 Vácrátót, Hungary; 2 GINOP Sustainable Ecosystems Group, MTA Centre for Ecological Research, Klebelsberg Kuno u. 3, 8237 Tihany, Hungary; 3 Department of Plant Systematics, Ecology and Theoretical Biology, Institute of Biology, Eötvös Loránd University, Pázmány P. stny 1/C, 1117 Budapest, Hungary

**Keywords:** Aboveground biomass, Climate change, *Conyza canadensis*, Phenotypic plasticity, Species interaction

## Abstract

During environmental change, invasive species may be favored by increased resource input or reduced resource use of the resident community. Plasticity in certain plant traits of invasive species may be one possible mechanism behind their ability to quickly exploit unused resources. We tested whether rainfall manipulations (severe drought, moderate drought, watering) alter the growth and reproductive success of the invasive annual *Conyza canadensis*, and if it translates into a change in the abundance of the species in a semiarid perennial grassland in Central Hungary. Overall, *C. canadensis* exhibited greater individual performance and higher abundance in drought plots than in control and watered plots. At individual level, plants showed the strongest response to moderate drought: they grew 2.5-times taller than in control and watered plots, and produced twice and 2.5-times more seeds than in watered and control plots, respectively. Reproductive phenology was advanced in response to rain exclusions. Although severe drought caused 40% mortality, the cumulative performance of *C. canadensis*, expressed as plot-level aboveground biomass, was consistently greater in severe drought plots than in control and watered plots throughout the 3 years of the study. The higher performance of *C. canadensis* in drought plots is most likely due to the decreased abundance and, thus, decreased competitive effect of previously dominant perennial grasses caused by the rain exclusions. We conclude that drier summers that suppress perennial grasses will favor this alien annual forb, and phenotypic plasticity in growth and reproduction may contribute to its invasion success.

**Electronic supplementary material:**

The online version of this article (10.1007/s00442-020-04711-y) contains supplementary material, which is available to authorized users.

## Introduction

Global environmental changes are likely to accelerate plant invasions (Dukes and Mooney 1999; Theoharides and Dukes 2007; Bradley et al. 2012), although the contraction of geographical range can also be projected for some recent invasive species (Bradley et al. 2009). Despite the intense research on the effects of climate change drivers on community invasibility and the success of invasive species, responses to precipitation changes have not been fully understood (Smith et al. 2000; Walther et al. 2009; Pfeifer-Meister et al. 2016; Thomason and Rice 2017; Gill et al. 2018). To improve our knowledge in this field is particularly important in arid and semiarid ecosystems, where water availability has a prominent role in determining plant diversity, primary production, and community stability (Rutherford 1980; Bai et al. 2004; Seddon et al. 2016).

The theory of fluctuating resource availability suggests that a plant community becomes more susceptible to invasion when there is an increase in resource availability (Davis et al. 2000). This can particularly be expected for annual invasive species, which may have an ability to exploit resources earlier, more rapidly or to a greater extent than native perennials (Leffler et al. 2011). Consistent with the fluctuating resource theory, a number of studies on invasive annual plants have shown that natural or experimental increase in rainfall facilitated invasion, while drought decreased the success of alien species (Hobbs and Mooney 1991; Dukes and Mooney 1999; Suttle et al. 2007; Har-Edom and Sternberg 2010; Suazo et al. 2012). Alternatively, alien species with rapid resource acquisition may be favored when drought causes plant mortality in the resident native vegetation, or limits the growth or reproduction of the resident species, which cannot immediately recover when drought stress is alleviated (Jiménez et al. 2011; Diez et al. 2012; Manea et al. 2016). Sheppard et al. (2012) suggested that climate change including extreme events is more likely to influence plant invasions indirectly via the impacts on native vegetation than by direct effects on the invasive species themselves.

Beside rapid evolutionary adaptation (e.g. Nguyen et al. 2016), the other important mechanism that allows invaders to establish and spread in a wide range of environments is phenotypic plasticity (i.e. the ability of a single genotype to express different phenotypes under different environmental conditions; Daehler 2003; Richardson and Pyšek 2006; Clements and Ditomaso 2011). A meta-analysis using data from invasive and phylogenetically related non-invasive species pairs showed that invasive species had consistently greater phenotypic plasticity than co-occurring non-invasives (Davidson et al. 2011). However, this higher plasticity was only sometimes associated with a fitness benefit over non-invasive species, partly due to the limited availability of accurate fitness data. This highlights the need to explore plasticity of traits that are directly related to fitness, such as vegetative biomass, seed biomass and number, and plant survival (Violle et al. 2007). Nevertheless, functional traits that impact fitness indirectly—for example reproductive phenology, which influences reproductive output—may also be crucial in the context of climate change and plant invasion. Wolkovich and Cleland (2011) predict that high phenological plasticity may enhance the success of invasive species as climate change increases variability in temperature and precipitation, which results in extended growing seasons. On the other hand, the population of the same alien species that exhibits constrained, genetically fixed reproductive phenology may be unable to respond opportunistically to unpredictable resource availability (Dyer et al. 2012). Several greenhouse or common garden experiments have shown phenological variation within or between populations of alien annual species in response to water availability (e.g. Dyer et al. 2012, 2016; Latimer et al. 2019). However, only few studies have investigated so far the phenological responses of invasive annuals to precipitation changes in field experiments in natural habitats (e.g. Prevéy and Seastedt 2014).

Horseweed (*Conyza canadensis* (L.) Cronq., Asteraceae), the selected species for our study, is a winter or summer annual herb native to North America, which has become a cosmopolitan weed (Weaver 2001; Bajwa et al. 2016) and the most widely distributed naturalized plant species in temperate regions (Pyšek et al. 2017). It is a ruderal species, which is among early colonizers during the secondary succession of disturbed habitats, such as abandoned agricultural fields and forest clear-cuts, but it usually fails to hold dominance in the later stages of succession (Keever 1950; Numata 1969; Weaver 2001). This species is frequently found in vineyards, along roadsides and railways (Weaver 2001), but can also invade natural sand grasslands and forests (Török et al. 2003; Djurdjević et al. 2011). Thus, *C. canadensis* is an ideal species to investigate whether drought directly decreases the abundance of an alien annual weed in semiarid grasslands. Or, alternatively, it can benefit from resource pulse ensuing from the reduced growth or reproduction of the resident dominant native species due to drought.

The objectives of this study were to assess: (1) how field-scale rainfall manipulations in a semiarid perennial grassland affect the growth and reproductive success of the invasive annual plant, *C. canadensis*, and (2) if it translates into a change in the abundance of the species. We hypothesized that plants show phenotypic plasticity in growth and reproductive traits in response to rainfall treatments, and this leads to altered abundance of *C. canadensis*. The drought response of various traits of *C. canadensis* has already been reported in pot experiments (Tremmel and Peterson 1983; Shrestha et al. 2010; Sheppard et al. 2012), but experimental evidence under field conditions is lacking.

## Materials and methods

### Study site and rainfall manipulation experiment

The study site is located in Central Hungary, near the village Fülöpháza (46° 52′ N, 19° 25′ E; 108 m above sea level) in the Kiskunság National Park. The region has temperate continental climate with sub-Mediterranean influences. Mean annual temperature is 10.4 °C, and mean annual precipitation is 505 mm for the study site (1961–1990; Kovács-Láng et al. 2000). The soil is nutrient-poor, coarse-textured calcareous sandy soil with ca. 11% CaCO_3_ and < 1% humus content in the upper 30-cm layer, and low water-holding capacity (Kovács-Láng et al. 2000). This extreme water and nutrient regime of the soil amplifies summer droughts typical in July and August. Mediterranean effects on the climate associated with drier summers are projected to become stronger in the future (Kis et al. 2017). In summer, the maximum number of consecutive dry days, the mean length of dry spells, and the total number of dry days are all forecasted to increase by the end of the twenty-first century (Pongrácz et al. 2014).

The studied vegetation is a semiarid, open perennial sand grassland, characterized by the dominance of two perennial bunchgrasses, *Festuca vaginata* Waldst. and Kit. ex Willd. and *Stipa borysthenica* Klokov ex Prokudin. The alien *Conyza canadensis* is a permanent member of the community as a summer annual forb, and is considered a disturbance-resistant pioneer species in open sand grasslands (Csecserits and Rédei 2001). This species usually germinates in April, and begins to flower in July at the study site.

In 2015, we set up a field experiment, where experimental units were 3 m × 3 m plots with a 50-cm buffer strip along the inner margin at each side of the plot. Thus, the effective sampling area was 2 m × 2 m. Plots were arranged in a completely randomized block design including three treatments (see below) and a control (ambient rainfall) in six replicates (six blocks, each block containing one plot of each treatment). Treatments included severe drought from late June to late August (ca. two months), moderate drought from late July to late August (ca. one month), and watering as one event of ca. 25 mm at around the 20th day of each month from May to August (i.e. 100 mm per year, which corresponds to about 20% increase over the long-term yearly average). Due to the relatively high uncertainty of the projections for changes in summer precipitation in the future (Pongrácz et al. 2014), these treatments covered the full range of precipitation changes from no drought (watering treatment) to severe (two-month) drought. At the onset of this study on *C. canadensis* (late May 2016), treatment plots had already underwent 1 year of rainfall manipulations (in 2015). Treatments continued in subsequent years (2016–2018) with similar timings (see Table 1 for exact dates of treatments in 2015 and 2016).

**Table 1:** Treatment periods in 2015 and 2016, precipitation (total sum) and daily average volumetric soil water content (SWC, %) between 1 May and 31 August 2015 and 2016 (i.e. during the period of the year covering each treatment), and the percentage cover (%) of the two dominant perennial grasses (*Festuca vaginata* and *Stipa borysthenica)* in the experimental plots in June 2015 and 2016 (before the current-year drought treatments), and September 2016 and 2018 (after finishing the current-year experimental treatments). SWC and cover data are treatment mean ± SE

	Treatment			
	Control	Severe drought	Moderate drought	Watering
Treatment period in 2015	–	23.06–25.08	22.07–25.08	18.05, 22.06, 21.07, 25.08
(Number of days)	(–)	(63)	(34)	(4 distinct)
Rainfall in 2015 (mm)	196.0	74.2	100.6	294.5
SWC (%) at 0–30-cm depth in 2015	4.0 ± 0.1	3.2 ± 0.1	3.4 ± 0.1	4.3 ± 0.1
Treatment period in 2016	–	23.06–25.08	20.07–25.08	25.05, 22.06, 21.07, 25.08
(Number of days)	(–)	(63)	(36)	(4 distinct)
Rainfall in 2016 (mm)	346.4	159.8	291.0	447.6
SWC (%) at 0–30-cm depth in 2016	5.0 ± 0.1	4.2 ± 0.2	4.6 ± 0.1	5.2 ± 0.1
Cover (%) of *F. vaginata* and *S. borysthenica* in June 2015	15.0 ± 2.3	13.5 ± 2.7	13.7 ± 2.4	18.9 ± 0.7
Cover (%) of *F. vaginata* and *S. borysthenica* in June 2016	17.6 ± 2.1	2.8 ± 0.6	10.0 ± 1.6	25.0 ± 2.1
Cover (%) of *F. vaginata* and *S. borysthenica* in September 2016	18.8 ± 2.2	2.3 ± 0.7	10.1 ± 2.0	29.0 ± 2.0
Cover (%) of *F. vaginata* and *S. borysthenica* in September 2018	18.3 ± 1.8	2.3 ± 0.6	10.2 ± 2.1	25.9 ± 1.6

In drought plots, we excluded rain using fixed, transparent polyethylene roofs, which is one of the most frequently used methods to impose experimental drought in water manipulation experiments (Hoover et al. 2018). In our experiment, transparent roofs transmitted 82.6% of incoming photosynthetic active radiation at noon in a clear July day. This value is consistent with the previous studies which reported that roofs made of the same material transmitted 75–93% of peak daytime photosynthetic photon flux density, and that did not limit plant growth (Fay et al. 2000; English et al. 2005). Watering treatment was applied using sprinklers at 1-m height, in a 1 m × 1 m grid. Side curtains were used to prevent rain from entering drought plots from the side, or to prevent irrigation water from falling into the plots neighboring watered plots.

Air temperature was measured at 20-cm height, and volumetric soil water content (SWC, %) was recorded at 0–30-cm depth (i.e. averaged over the soil profile) in each plot by permanent temperature and moisture sensors (Sensirion SHT75 and Campbell CS616, respectively) connected to a data logger. Precipitation was measured with rain gauges (Davis DS7852) at 30-cm height. In addition, a standard meteorological station is operating next to the study site since 2001.

### Background conditions for the studied *C. canadensis* plants: precipitation, soil water content, and the abundance of dominant grasses

In 2015, annual precipitation (523 mm) was close to the average of the previous 14 years (589 mm). In this year, severe and moderate drought treatments excluded 23% and 18% of ambient annual precipitation, respectively; while watered plots received 19% more rainfall than control plots (Table 1). In 2016, which was a wet year, rainfall amounts excluded from severe and moderate drought plots were 25% and 7% of the ambient annual precipitation (742 mm), respectively. Due to the relatively high amount of natural rainfall in 2016, the ca. 100-mm watering treatment in this year increased yearly precipitation by 14% compared to control.

Overall, rain exclusions had negative effects, while watering exerted a positive influence on the soil water content averaged for the period of the year covering each treatment (1 May–31 August) in both 2015 and 2016 (Table 1). Interestingly, in May 2016, SWC was significantly higher in severe drought plots (6.1%) than in control and watered plots (5.2% and 5.0%, respectively).

The cover of *F. vaginata* and *S. borysthenica* in June 2015 (i.e. prior to rain exclusions in the first year of the experiment) was similar in control and treatment plots (Table 1). Drought treatments applied in 2015 decreased drastically the cover of these two perennial grasses, which remained low in June 2016 (i.e. prior to the application of current-year drought treatments). Similar differences were found between control and drought plots in the cover of *F. vaginata* and *S. borysthenica* in September 2016 and 2018, after finishing the current-year rain exclusions. In watered plots, the cover of these two dominant species was about one and a half times higher than that in control plots in June and September 2016, and in September 2018.

### Field sampling and data collection

In June 2016, ten individuals of *C. canadensis* were randomly selected and marked for repeated measurements within the 4-m^2^ sampling area of each plot. In a few plots, where less than ten but at least five individuals occurred, all plants were examined (one watered and one control plot with less than five individuals per plot were omitted). Plant mortality rate per plot was determined as the percentage of marked individuals that died by the end of August (end of the experimental treatments). For the individuals that survived until the end of treatments (referred to as alive individuals hereafter), we measured the maximum vegetative shoot height (stretched length of the shoot beneath inflorescence) according to Cornelissen et al. (2003).

The length and width (in the middle of the inflorescence) of fully developed inflorescence were measured at the end of August or early September 2016. The number of capitula per plant was estimated using a linear regression equation (*r*^2^ = 0.81) between the length × width of inflorescence and the number of capitula determined on additional sixty-five individuals outside, but close to the experimental plots. Twenty capitula with developing achenes were collected from additional individuals outside the plots, and the number of seeds (achenes) per capitulum was counted to determine the average number of seeds in a capitulum. The seed number of plants growing in the experimental plots was determined as the number of capitula multiplied by the average number of seeds per capitulum (46 seeds). This estimation allowed us to determine seed number per plant non-destructively using relatively large sample size (i.e. each marked alive individual per plot). Similar estimation of seed or flower production is commonly used in field experiments to avoid destructive sampling within the experimental plots (e.g. Llorens and Peñuelas 2005; Pieper et al. 2011).

Reproductive phenology of marked individuals was monitored weekly or biweekly from the end of August to the middle of October 2016. The phenological stages of capitulum development were grouped into two categories: (1) flowering stage, which included unopened and opened capitula, and (2) fruiting stage, which included capitula with (developing or mature) achenes and capitula that had shed their achenes. At each date, we calculated the percentage of individuals having capitula in the flowering and fruiting stage, separately, relative to the total number of marked alive individuals per plot.

The density of *C. canadensis* per plot was assessed by counting individuals in a 1 m × 1 m quadrat in each plot in late May 2015 and 2016, prior to the onset of the current-year experimental treatments. This quadrat size was chosen based on the previous studies that used quadrats of similar or smaller size to determine the density of *C. canadensis* (Steckel et al. 2006; McCauley et al. 2018). To evaluate the performance of *C. canadensis* at plot level without destructive sampling, the aboveground biomass of this species was determined as follows. The canopy cover of *C. canadensis* was visually estimated in four 1 m × 1 m quadrats in each plot in June and September (i.e. before and after drought treatments) each year between 2016 and 2018. Data of the four quadrats were averaged for each plot. Cover data were converted to aboveground biomass using a linear regression equation (*r*^2^ = 0.96) between the visually estimated cover and aboveground biomass of *C. canadensis* obtained in additional twelve 1 m × 1 m quadrats outside the experimental plots in September 2016. The aboveground biomass of *C. canadensis* was harvested, dried at 60 °C for 48 h, and then weighed.

### Statistical analysis

General linear mixed models with treatment as a fixed effect and block as a random factor were conducted for maximum vegetative shoot height, seed number per plant, the percentage of individuals having capitula in the flowering and fruiting stages (at the end of August and September separately), the density of individuals, and mortality rate. If necessary, data were square-root transformed to improve normality and homoscedasticity assumptions (Quinn and Keough 2002). For post hoc comparison of means, Tukey’s HSD tests were used. To assess the effect of shoot height on seed number after controlling for the effect of treatments, shoot height was also included in the model as a continuous predictor variable, and the partial correlation coefficient (*R*) was calculated.

Two-way repeated measures ANOVA was used for square-root transformed data of plot-level aboveground biomass with treatment as a fixed between effect, and month (i.e. June and September) as a repeated measures (within) effect, for each year (2016–2018) separately. Tukey’s HSD tests were used to check pairwise differences between treatments within each month.

For each analysis, the TIBCO Statistica software (TIBCO Software Inc. 2018) was used, and differences were considered significant at *P* < 0.05.

## Results

**Fig. 1:**
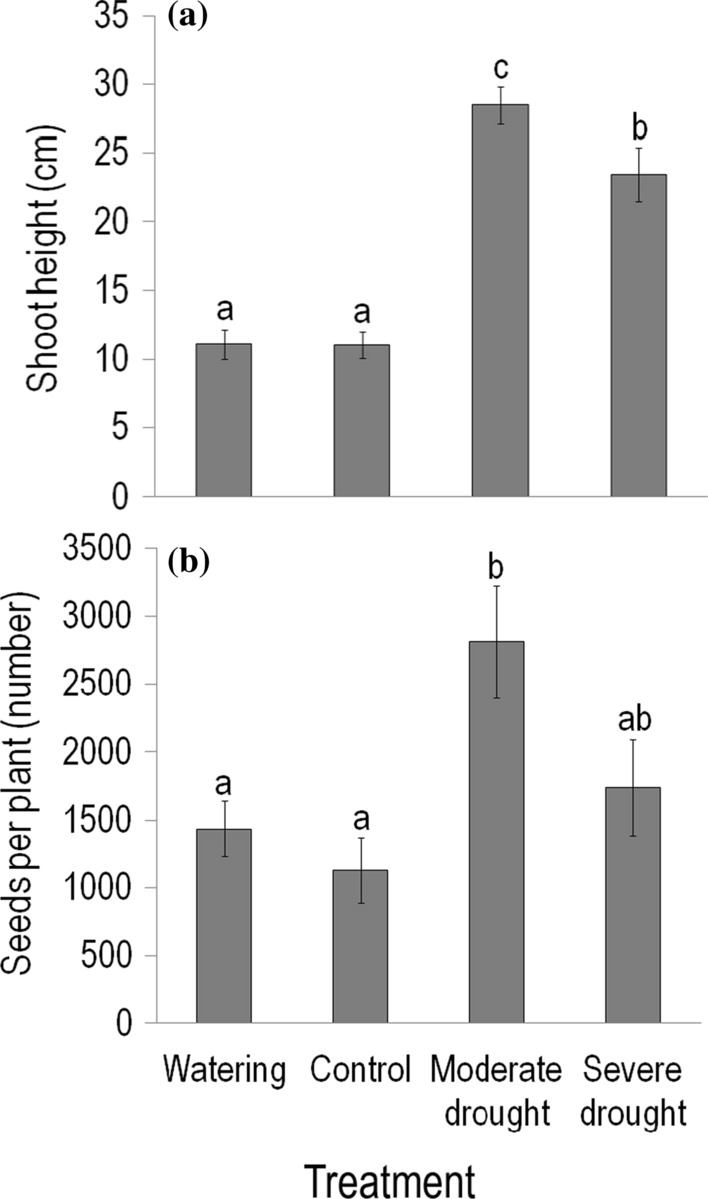
Effects of rainfall manipulations on **a** the maximum vegetative shoot height (cm) and **b** seed number per plant of *Conyza canadensis* growing in the plots of the field experiment in 2016. Values are treatment mean ± SE. Results of Tukey’s HSD tests following general linear mixed models are shown. Different letters above the bars indicate significant (*P* < 0.05) differences between treatments

Rainfall manipulations had a strong effect on almost all plant response variables studied for *C. canadensis* growing in the plots of the field experiment (Online Resource Table S1). Both maximum vegetative shoot height and seed number per plant were the highest for plants growing in moderate drought plots (although the difference in seed number was only marginally significant between severe and moderate drought plots, *P* = 0.079; Fig. 1a, b). Shoot height in severe drought plots was also greater than in control and watered plots (about twice), while seed number per plant was statistically similar in control and these two treatment plots. When controlling for the effect of treatments, seed number per plant showed a positive partial correlation with shoot height (*R* = 0.45, *P* < 0.0001).

**Fig. 2:**
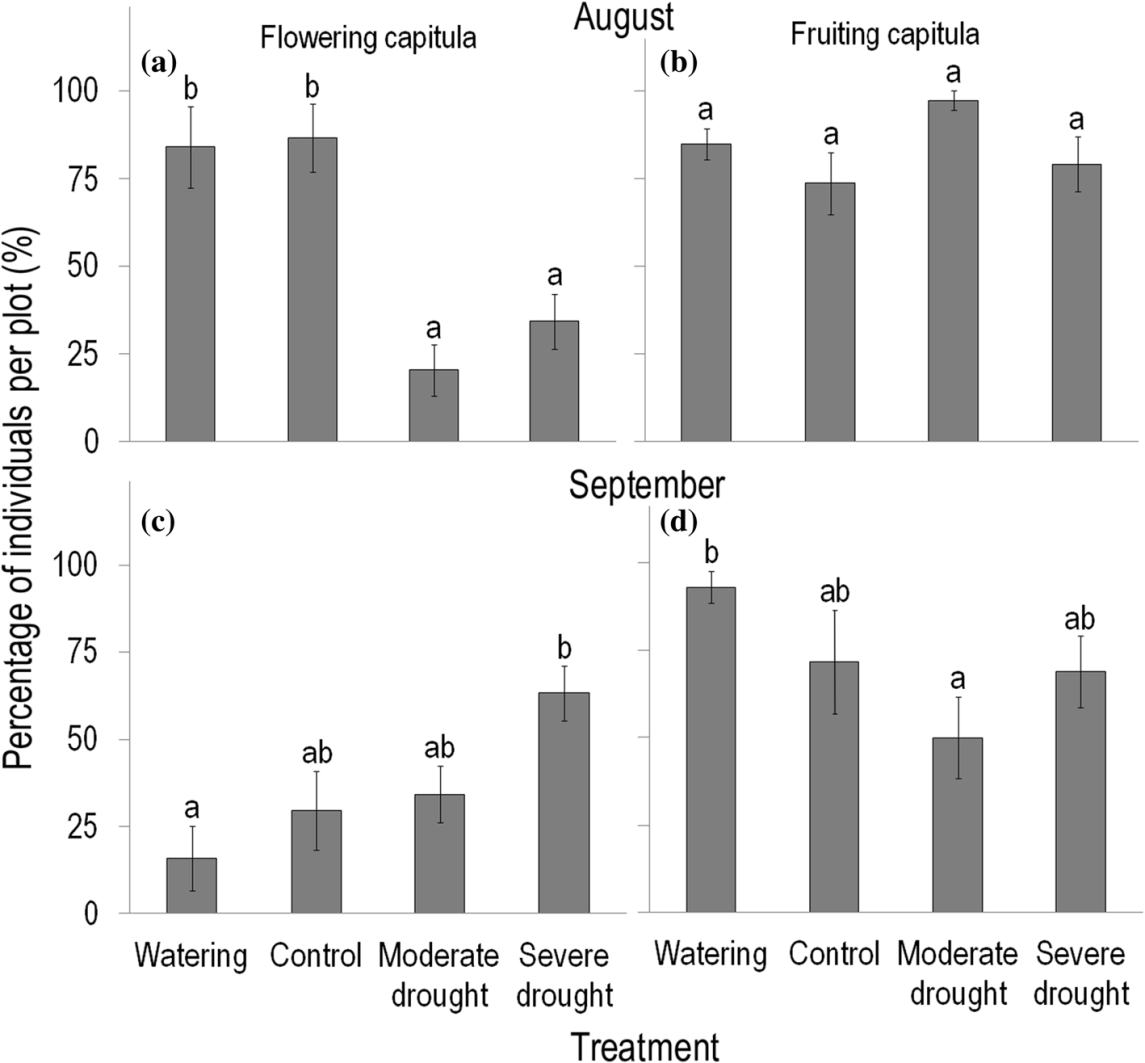
Effects of rainfall manipulations on the percentage of *Conyza canadensis* individuals having capitula in the **a**, **c** flowering stage and **b**, **d** fruiting stage at the end of August (top panels) and at the end of September (bottom panels) 2016. These percentages were calculated relative to the total number of marked individuals of *C. canadensis* per plot that survived until the end of treatments. Flowering capitula denote unopened and opened capitula, while fruiting capitula refer to capitula with (developing or mature) achenes, and capitula that had shed their achenes. Values are treatment mean ± SE. Statistical tests were applied for flowering and fruiting capitula separately within a month. Statistical tests and the indication of significant (*P* < 0.05) differences between treatments are the same as described in the legend of Fig. 1

Regarding reproductive phenology, flowering was more responsive to rainfall manipulations than fruiting (Online Resource Table S1, Fig. 2a–d). At the end of August, just after the cessation of treatments, the percentage of individuals having flowering capitula was more than 80% in control and watered plots, whereas only 20% and 34% in moderate and severe drought plots, respectively (Fig. 2a). At the same time, the percentage of individuals having capitula in the fruiting stage was similarly high (74–97%) in control and each treatment plot (Fig. 2b). A month later, by the end of September, the percentage of individuals having flowering capitula changed markedly relative to that found in August: it almost doubled in severe drought plots, where it exceeded the value observed in watered plots (and also control plots with marginally significant difference, *P* = 0.086; Fig. 2c). This was because during September, new capitula emerged on 31% of plants in severe drought plots; while in control and watered plots, the percentage of individuals having flowering capitula decreased to 30% and 16%, respectively.

**Fig. 3:**
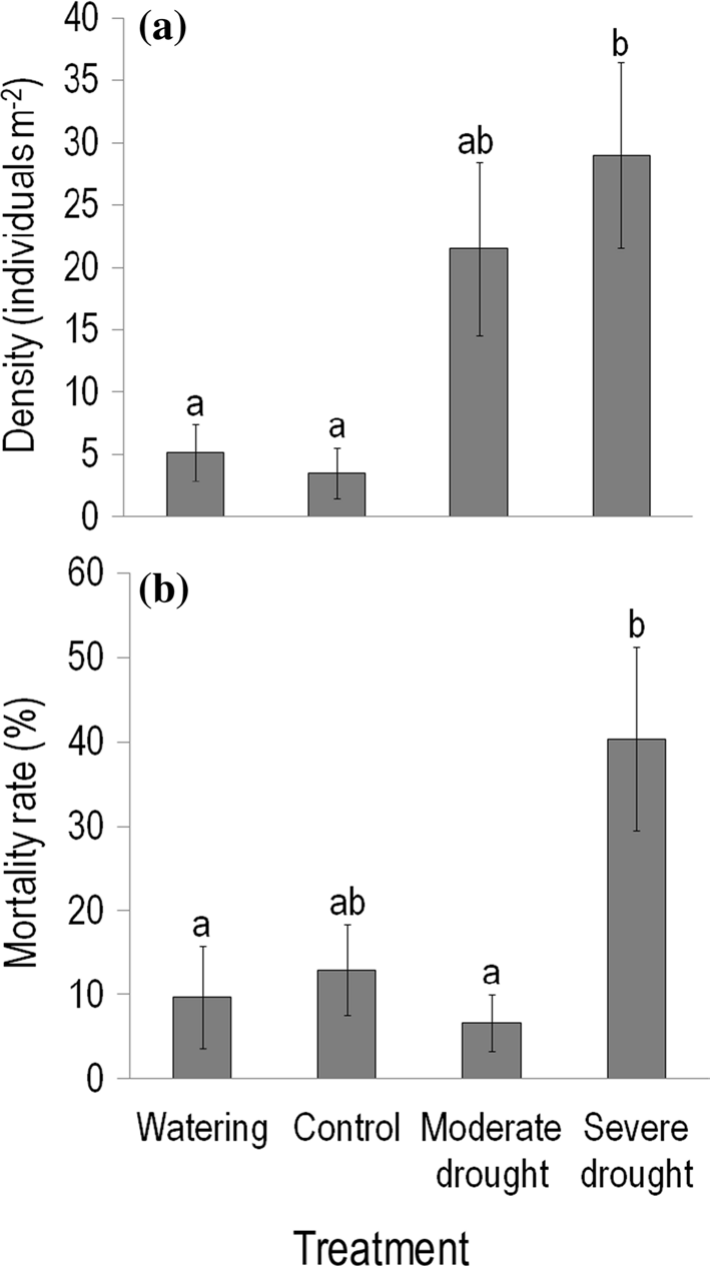
**a** Density of individuals in late May (prior to the current-year experimental treatments) and **b** mortality rate at the end of August (just after finishing the treatments) for *Conyza canadensis* growing in the plots of the field experiment in 2016. Values are treatment mean ± SE. Statistical tests and the indication of significant (*P* < 0.05) differences between treatments are the same as described in the legend of Fig. 1

The density of *C. canadensis* individuals in May 2015 (i.e. pre-treatment values in the first year of the experiment) did not differ significantly between control and treatment plots (*P* = 0.49). In contrast, in May 2016, before starting the current-year experimental treatments, plant density in severe drought plots exceeded that detected in control and watered plots, about eight times and more than five times, respectively (Fig. 3a). Compared to control plots, plant density was also greater (about six times) in moderate drought plots with marginally significant difference (*P* = 0.076). By the end of treatments, plants growing in severe drought plots experienced the highest (40%) mortality rate, with marginally significant difference from control (13%, *P* = 0.058; Fig. 3b). The greatest (sixfold) difference was found between severe and moderate drought plots.

**Fig. 4:**
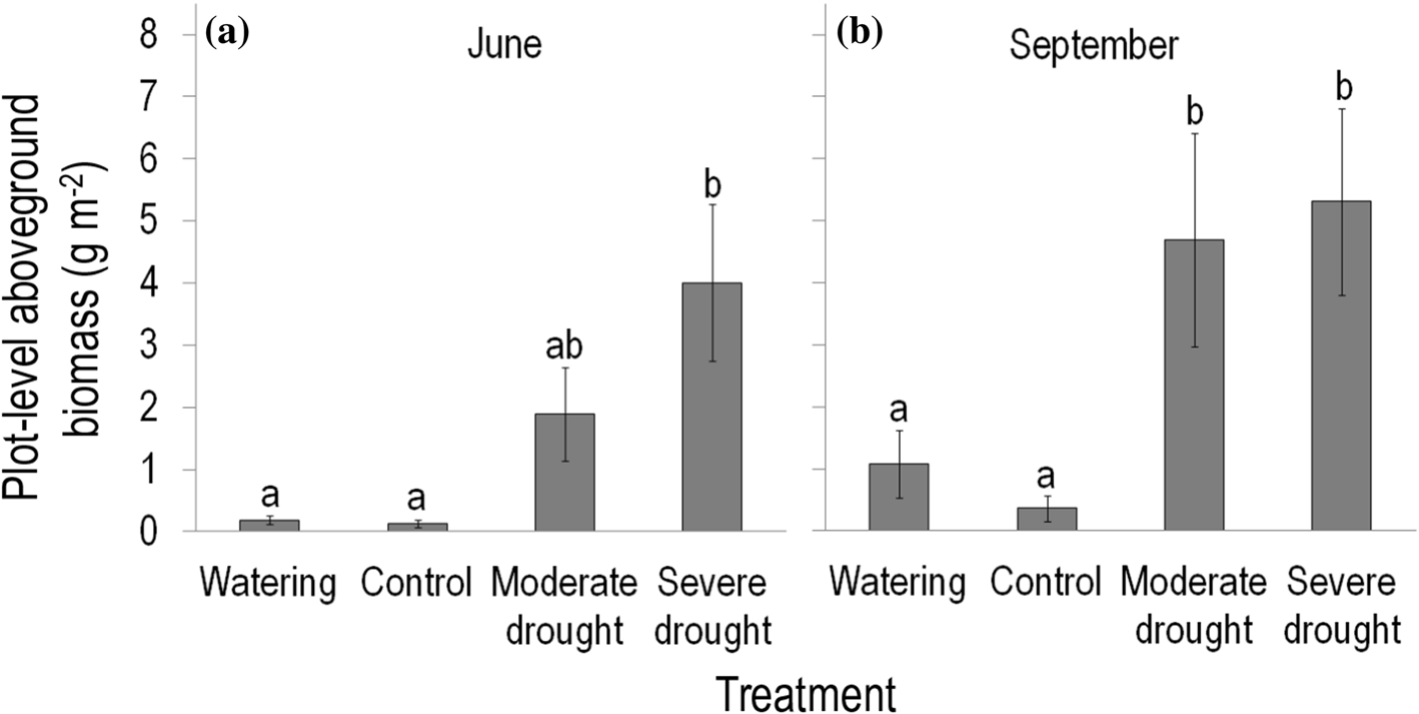
Plot-level aboveground biomass (mean ± SE) of *Conyza canadensis* growing in the plots of the field experiment in **a** June (before the current-year drought treatments) and **b** September (after finishing the current-year experimental treatments) 2016. Results of Tukey’s HSD tests in June and September separately, following two-way repeated measures ANOVA are shown. Different letters above the bars indicate significant (*P* < 0.05) differences between treatments

The plot-level aboveground biomass of *C. canadensis* was higher in severe drought plots (4.0–5.3 g m^−2^) than in control and watered plots (≤ 1.1 g m^−2^) both preceding and following the current-year drought treatments (in June and September 2016, respectively; Fig. 4a, b). Consistently, similar differences were found in the subsequent two years (2017 and 2018; Online Resource Fig. S1a–d). In the moderate drought plots, aboveground biomass showed intermediate values except in September 2016, when it also exceeded those estimated in control and watered plots (Fig. 4b).

## Discussion

### Individual plasticity in response to rainfall manipulations

Shoot growth, seed production and reproductive phenology of *C. canadensis* plants provided evidence in favor of our hypothesis, that *C. canadensis* growing in the experimental plots exhibited phenotypic plasticity in the studied traits in response to rainfall treatments. Surprisingly, plants growing in drought plots showed enhanced growth compared to individuals growing in control and watered plots. This positive response was strongest for plants that experienced one-month drought, and these individuals also had higher seed production. Thus, at individual level, *C. canadensis* exhibited the greatest vegetative and reproductive performance in the environment created by moderate (1-month) drought.

In contrast with our results, experimental drought decreased plant height compared to high water availability for *C. canadensis* in pot experiments (Tremmel and Peterson 1983; Shrestha et al. 2010), and for nine out of ten annual species typical of Mediterranean grasslands in a common garden experiment (Pérez-Ramos et al. 2019). The most likely explanation for these apparently contradictory results is that *C. canadensis* may have benefited indirectly from the 1-month rain exclusion of the previous year (2015). This species likely experienced better resource (particularly water) availability due to the decrease in the abundance (and thus the competitive effect) of the two dominant perennial grasses, which did not recover by June 2016 (Table 1). However, the lower shoot height and seed production in severe than in moderate drought plots indicate that such indirect benefit of drought was reduced by the direct limitation of the current-year (2016) 2-month rain exclusion on the performance of *C. canadensis*. Severe drought treatment was not only longer than moderate drought treatment, but also commenced 1-month earlier, thus probably at a more susceptible stage of plant growth. An indirect advantage of rain exclusion was also reported for the native subordinate winter annual grass, *Secale sylvestre* Host in the same precipitation manipulation experiment (Mojzes et al. 2018). In line with our results, Poll (2007) found that *C. canadensis* individuals that were planted into the non-competition plots, where all vegetation was removed before planting, grew taller than those planted into the competition plots (intact native vegetation).

In our study, the positive relationship between shoot height and seed number indicates that plants which experience better resource availability can invest more assimilates to both vegetative growth and seed production. Regehr and Bazzaz (1979) also reported that the seed production of *C. canadensis* was proportional to mature plant height. Furthermore, previous results showed that the mean dispersal distance of *C. canadensis* seeds proportionally increased with the release height of seeds (Dauer et al. 2006). Thus, in our study, taller plants in drought plots may have a dispersal advantage: a potential for increasing the rate of spread by establishing new colonies (i.e. new foci of invasion; Cronk and Fuller 2013).

The reproductive phenology of *C. canadensis* also showed plasticity in response to rainfall manipulations. The high proportion of individuals having capitula in the fruiting stage and the parallel low proportion of plants having flowering capitula in drought plots at the end of the experimental treatments suggest that rain exclusions advanced the flowering phase. This response can be interpreted as a plastic drought escape strategy, when plants develop rapidly and reproduce before drought conditions become prevalent. Water availability is considered to be the most important environmental cue to induce such a drought escape response (Kooyers 2015). This strategy may ensure successful reproduction, thus contributing to the persistence or spread of *C. canadensis* in drier summers projected for the future (Pongrácz et al. 2014; Kis et al. 2017). In agreement with our results, Mooney et al. (1986) observed earlier flowering in most native annual species in a serpentine rock grassland in the two dry years compared with the non-drought year of their study period. Similarly, in the experiment of Dyer et al. (2012), the invasive annual *Bromus tectorum* L. set seed earlier under low-water treatment than under high-water condition. By contrast, experimental drought induced a delay in the peak fruiting date of ten annual species in a common garden experiment (Pérez-Ramos et al. 2019). In our study, however, a month after the cessation of treatments, we found an unexpected emergence of new capitula on a number of plants growing in severe drought plots. This indicates that these plants did not finish their reproductive phase by the end of the experimental drought, but started to flower again. This phenomenon may be due to the favorable weather of September 2016, when the monthly average temperature was 1.4 °C higher than the mean (16.0 °C) of the previous 15 years (2001–2015), and at the same time, precipitation (48.2 mm) was close to the 15-year monthly average (54.2 mm). The capacity of *C. canadensis* for secondary flowering allowed additional reproduction when drought stress was alleviated. The lack of similar second phase of flowering in control and watered plots may likely be attributed to the higher abundance of dominant perennial grasses (Table 1), which competed for resources more successfully than the annual *C. canadensis.* Consistent with our result, Bergmeier (1998) reported a second, minor peak of flowering for many annual species of Mediterranean phrygana vegetation subsequent to ample rain in late spring, but such re-flowering failed in a year when no late-spring precipitation was recorded. Dyer et al. (2012) found a similar response to high-water treatment in the mesic population of *Bromus tectorum*: 38% of individuals developed new tillers after the initial harvest, and produced additional seeds.

### Population responses to rainfall manipulations

Consistent with our hypothesis, the higher individual performance (seed production and/or shoot height) of *C. canadensis* in response to rain exclusions led to higher abundance, expressed as plot-level aboveground biomass of this species relative to control and watering treatment. Each variable studied for *C. canadensis* at population level, i.e. the density of individuals, mortality rate, and plot-level aboveground biomass, responded most strongly to severe drought treatment.

The greater density of individuals in severe drought plots than in control and watered plots before the onset of experimental treatments in 2016 may show the indirect benefit of the previous-year (2015) severe drought treatment for *C. canadensis*. The two-month rain exclusion applied in 2015 decreased the abundance of the two dominant perennial grasses compared to control and watering treatment, and the differences remained by June 2016 (Table 1). This may have provided less competitive environment, thus better water availability for the seedling survival and early growth of *C. canadensis* in spring 2016. This interpretation is supported by the higher soil water content in severe drought plots (6.1%) than in control and watered plots (5.2% and 5.0%, respectively) in May 2016 (see also Methods). Consistent with our results, the seedling survival of *C. canadensis* was highly susceptible to interspecific competition in a greenhouse experiment (Tremmel and Peterson 1983).

At the adult stage of *C. canadensis*, we found the highest mortality rate in severe drought plots, which may reflect the direct negative effect of the 2-month drought on plant survival. However, those plants that survived the two-month rain exclusion grew taller than the individuals growing in control and watered plots. In another experiment where *C. canadensis* rosettes were transplanted to (4-year- and 17-year-old) abandoned fields, the probability of survival to reproduction increased by resource (nutrients and water) addition, but was reduced by interspecific competition with neighboring plants (Thébaud et al. 1996). Our results together with those cited above show that *C. canadensis* may be both relatively drought sensitive, and highly responsive to competitive interactions with neighbors in the surrounding vegetation.

Despite the high mortality rate of *C. canadensis* in severe drought plots, the cumulative performance of this species, expressed as plot-level aboveground biomass, was higher in these plots than in control and watered plots, and these differences were consistent throughout the 3 years (2016–2018). This indicates that at population level, the environment altered by the recurrent two-month drought may be the most favorable for the persistence of *C. canadensis* in open sand grasslands.

Our results suggest that in sand grasslands, the abundance and persistence of *C. canadensis* are more strongly determined by changes in the competitive interactions with the dominant perennial grasses in response to altered precipitation amount than by the direct impact of precipitation change. These results are consistent with the previous experiments where competition with native plants in the intact local plant community strongly reduced the aboveground biomass of *C. canadensis* relative to plots where all native plants were removed (Poll 2007; Har-Edom and Sternberg 2010).

## Conclusions

Our study showed that experimental reduction in precipitation indirectly enhanced the performance of the invasive annual *C. canadensis* in a semiarid perennial grassland. Both the shoot growth and seed production of this species exhibited the greatest response to moderate (1-month) drought. However, the environment created by severe (2-month) drought induced the strongest cumulative positive response of *C. canadensis*, expressed in plot-level aboveground biomass, and this was consistent in three consecutive years. In addition, advanced flowering in response to rain exclusions may ensure successful reproduction of this species before drought stress becomes prevalent. Based on our results, the substantial decrease in summer precipitation projected for Hungary (Kis et al. 2017) may indirectly help *C. canadensis* to persist or even expand in open sand grasslands, and phenotypic plasticity in growth and reproduction may contribute to the invasive success of the species in such a changing climate.

## Electronic supplementary material

Below is the link to the electronic supplementary material.

Supplementary material 1 (PDF 177 kb)
