# Supplementary material for: Experimental drought indirectly enhances the individual performance and the abundance of an invasive annual weed
Source: Oecologia. 2020 Jul 17;193(3):571–81. doi: 10.1007/s00442-020-04711-y (PMC7406490; doi:10.1007/s00442-020-04711-y)
Supplement: Supplementary file 1 — Supplementary material 1 (PDF 177 kb) [file 442_2020_4711_MOESM1_ESM.pdf]

Electronic Supplementary Material

Title: **Experimental drought indirectly enhances the individual performance and the abundance of an invasive annual weed**

Authors: Andrea Mojzes*, Gábor Ónodi, Barbara Lhotsky, Tibor Kalapos, György Kröel-Dulay

*Corresponding author

Contact information for the corresponding author: MTA Centre for Ecological Research, Institute of Ecology and Botany, Alkotmány u. 2-4, H-2163 Vácrátót, Hungary; E-mail: mojzes.andrea@okologia.mta.hu

Tel.: +36 28 360122; +36 28 360147; Fax: +36 28 360110

Online Resource Table S1 ANOVA table for plant response variables studied for *Conyza canadensis* growing in the plots of the field experiment. The column $df_{Num; Den}$ includes the degrees of freedom of the numerator (MS predictor) and the denominator (MS error), respectively. Bold *P* values are significant at 0.05. Flowering and fruiting capitula (%) refer to the percentage of individuals having capitula in the flowering and fruiting stage, respectively. These percentages were calculated relative to the total number of marked individuals of *C. canadensis* per plot that survived until the end of treatments. Plot-level biomass denotes the plot-level aboveground biomass of *C. canadensis*. All variables were determined in 2016, except plot-level biomass, which was estimated each year between 2016 and 2018

Plant response variable (predictor)	$df_{Num; Den}$	F	*P*
Shoot height (cm)	3; 140	52.52	< **0.0001**
Seeds per plant (number)	3; 140	3.97	**0.0094**
Flowering capitula (%) in August	3; 13	20.96	< **0.0001**
Fruiting capitula (%) in August	3; 13	2.15	0.14
Flowering capitula (%) in September	3; 13	4.84	**0.018**
Fruiting capitula (%) in September	3; 13	3.38	0.051
Plant density (individuals m^{-2})	3; 15	6.49	**0.0050**
Mortality rate (%)	3; 13	5.46	**0.012**
Plot-level biomass (g m^{-2}) in 2016			
Treatment	3; 20	13.47	< **0.0001**
Month	1; 20	7.53	**0.013**
Treatment × Month	3; 20	0.60	0.62
Plot-level biomass (g m^{-2}) in 2017			
Treatment	3; 20	5.80	**0.0051**
Month	1; 20	0.88	0.36
Treatment × Month	3; 20	0.26	0.85
Plot-level biomass (g m^{-2}) in 2018			
Treatment	3; 20	7.68	**0.0013**
Month	1; 20	0.0030	0.96
Treatment × Month	3; 20	0.44	0.73

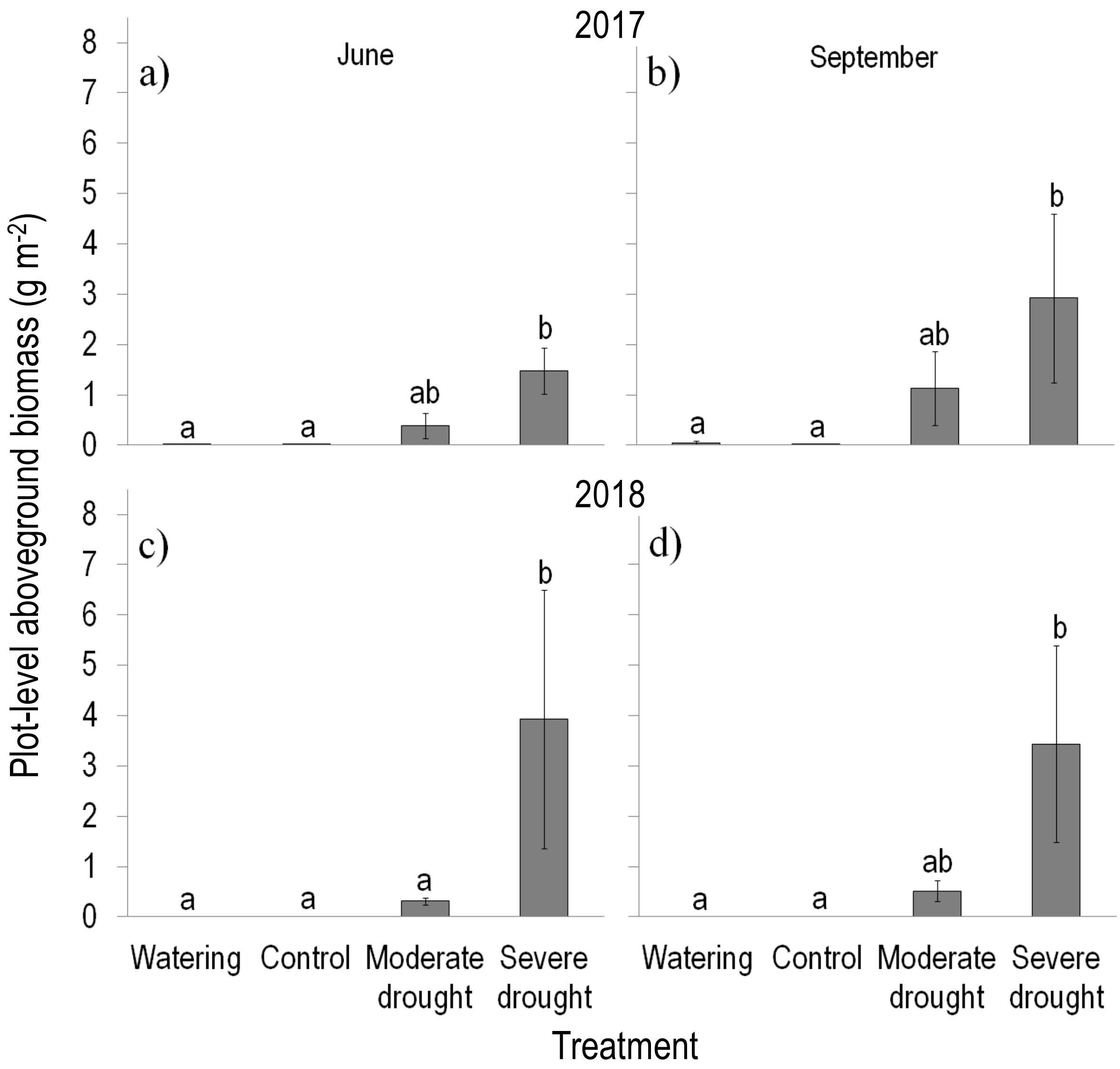

Online Resource Fig. S1 Plot-level aboveground biomass (means ± SE) of *Conyza canadensis* growing in the plots of the field experiment in a, c) June (before the current-year drought treatments) and b, d) September (after finishing the current-year experimental treatments) 2017 (top panels) and 2018 (bottom panels). For each year, results of Tukey's HSD tests in June and September separately, following two-way repeated measures ANOVA are shown. Different letters above the bars indicate significant ($P < 0.05$) differences between treatments
